# Investigation of the Role of Political Participation in Health: A Scoping Review

**DOI:** 10.1002/hsr2.72167

**Published:** 2026-05-17

**Authors:** Zahra Heydarifard, Mohammad Veisi, Majid Heydari, Iravan Masoudi Asl, Morteza Arab‐Zozani, Marzie Zarqi, Seyed Mehdi Emamian Fard

**Affiliations:** ^1^ Department of Health Services Management, School of Health Management and Information Sciences Iran University of Medical Sciences Tehran Iran; ^2^ National Center for Health Insurance Research Tehran Iran; ^3^ National Agency for Strategic Research in Medical Education Ministry of Health and Medical Education Tehran Iran; ^4^ Social Determinants of Health Research Center Birjand University of Medical Sciences Birjand Iran

**Keywords:** citizen science, health, health politics, political medicine, political participation

## Abstract

**Background and Aims:**

Political participation in health is crucial for shaping health policies and ensuring community engagement in decision‐making processes. This study explores the role of political participation in health by examining citizen science, political medicine, and other forms of public health involvement.

**Methods:**

This study was conducted as a scoping review based on the final framework (Peters et al.). The review process followed the JBI Manual for Evidence Synthesis and was reported in accordance with the PRISMA‑ScR checklist. A comprehensive search of PubMed, Web of Science, Scopus, ProQuest, and Google Scholar was carried out on February 19, 2023, using the finalized search strategy. Two reviewers independently screened titles, abstracts, and full texts, with disagreements resolved through discussion. Data were extracted using a standardized form. The final results were categorized using the STEEP framework (social, technological, economic, environmental, political) and complemented by SWOT analysis.

**Results:**

Of the 972 records identified, 282 duplicates were removed, and 617 abstracts and titles were excluded. Following full‐text screening, 58 studies were excluded, leaving 15 publications for analysis. The influence of various factors on political participation in health was assessed using STEEP and SWOT analysis. Findings indicate that fostering community health literacy, ensuring equitable access to political processes, and addressing socio‐economic determinants can enable policymakers to develop more inclusive and effective health policies.

**Conclusion:**

Political participation plays a vital role in shaping health outcomes. Strengthening strategies that enhance community involvement in health decision‐making is essential for achieving a more inclusive and equitable health system.

## 1 Introduction

Health policy is deeply interconnected with other social sectors, and in recent years, public engagement policies have been promoted as a tool for enhancing the accountability of participatory health care systems [1]. In this context, health policy is defined as: a set of actions (and even inactions) that affect organizations, personnel, services, financing arrangements, and stakeholders in the field of health care. It encompasses decisions made in both the public and private sectors and also includes external institutions that influence health [2]. Community engagement is defined by WHO as “a process of developing relationships that enable stakeholders to work together to address health‐related issues and promote well‐being to achieve positive health impact and outcomes” and is considered a fundamental component of a fair, rights‐based approach to health [3]. Since the 1990s, participation has been a central theme in health sector discourse, rooted in the primary health care framework introduced in the 1970s [4]. More recently, community engagement has regained prominence as a global priority, particularly in alignment with the Sustainable Development Goals (SDGs), which emphasize people‐centered, integrated health services as essential for achieving universal health coverage [5]. Political participation in health takes both contentious and deliberative forms. Protests over health care costs, as seen in some countries during austerity [6], contrast with citizen councils and participatory committees that foster dialogue on priorities and inequalities [7]. Together, they show the multifaceted nature of engagement, which is crucial for tackling unequal access, weak system responsiveness, and public skepticism in health crises [8, 9]. These challenges highlight the need for inclusive decision‐making.

Beyond improving the effectiveness of health programs, public participation in health care decision‐making is increasingly recognized for its role in fostering responsive policies, improving health care services, empowering communities, and reducing health inequalities [10, 11]. The importance of community participation in the health policy cycle was first highlighted at the World Conference on Health Promotion in Ottawa (1986), where community empowerment was identified as a cornerstone of success in health promotion efforts [12]. Similarly, the Alma‐Ata Declaration (1978) marked a significant shift in the conceptualization of health, prioritizing equity, participation, and the social determinants of health [13]. In 2025, the global agenda is redefined more comprehensively in the World Report on Social Determinants of Health Equity. In addition to emphasizing the impact of the distribution of power, financial resources, and opportunities on health equity, the report highlights participatory governance approaches and the inclusion of civil society in decision‐making, as well as coordinated action at both governmental and societal levels [14]. Political participation in public health takes diverse forms, ranging from voting and consultations to community mobilization, each shaping how health priorities are defined [2]. Illustrations such as public demonstrations over health care costs in OECD countries during austerity highlight the impact of collective action on affordability reforms [6], while citizen councils in European settings have been instrumental in addressing systemic inequities in health systems [7, 15]. Clarifying these forms is essential for understanding how public involvement influences health governance. Engagement policies, including participatory budgeting and advisory boards, aim to institutionalize community input and ensure responsiveness to local needs [3, 14].

In this regard, the WHO, at the 77th World Health Assembly, adopted a resolution under which Member States are required to cooperate in the implementation, strengthening, and continuation of regular and meaningful social participation in health decision‐making processes [16]. Social participation, as defined by the WHO, is closely associated with the concept of public involvement in health policy‐making; it is defined as the empowerment of individuals, communities, and civil society through inclusive participation in decision‐making processes across the entire policy cycle and at all levels of the system [16]. The International Labor Conference, in a resolution on decent work and the care economy, emphasizes the need to ensure workers' participation through social dialogue. The resolution also defines participation as a social responsibility in which governments, the private sector, and families must all be engaged. Likewise, the United Nations has called for a model of shared responsibility in the provision of care, aimed at safeguarding human rights [17]. As one of the influential determinants of health, an increasing body of evidence suggests that civic and political participation can positively affect health outcomes [10, 18, 19, 20]. Political participation and political activism refer to the activities through which citizens influence the selection of policymakers or the policies being implemented. Political activism emphasizes more direct involvement in shaping policy agendas, often occurring at the grassroots level [18]. On the other hand, an individual's health status can also affect the likelihood of their political participation. This bidirectional relationship is evident across different demographic and socioeconomic contexts [21, 22].

Political participation by groups such as women, citizens, and a diverse range of stakeholders in health sector planning and the development of responsive health strategies plays a crucial role in shaping health outcomes and policies [15, 23]. Incorporating varied perspectives, including both proponents and critics, can help identify potential solutions to prevent unintended policy consequences within complex health systems [24, 25]. Citizen science, which refers to the active participation of ordinary people in scientific processes, either through collaboration with professional researchers or by independently applying scientific methods, has increasingly been recognized as a promising pathway for advancing public health [26]. Studies highlight its potential to democratize health research, foster public engagement, and enhance health outcomes at the community level [27]. Furthermore, the relationship between health and political participation extends beyond individual engagement, influencing broader societal structures and governance [28]. This study aims to explore the effects of public political participation on health outcomes, assess the role of citizen engagement in health planning and policymaking, examine the impact of citizen science on public health, and provide insights into the bidirectional relationship between health and political engagement. By addressing these dimensions, the study seeks to offer a comprehensive understanding of the complex interplay between political participation and health, providing valuable insights for managers, policymakers, and researchers.

## 2 Hypotheses

Based on existing literature on the political and structural determinants of civic involvement in health governance, we propose the following hypotheses [6, 7, 8].

Higher levels of social capital and health literacy are positively associated with increased public participation in health‐related decision‐making processes.

Technological advancements, particularly the availability of digital health engagement platforms, enhance citizens' opportunities and likelihood to participate in health governance.

Political openness and institutional transparency are significant predictors of effective public engagement mechanisms within health systems.

## 3 Methods

### 3.1 Aim, Design, and Overall Framework

The present study was a scoping review. In this study, the general framework proposed by Arksey and O'Malley (2005), which was subsequently refined in two stages by Levac et al. (2010) and finally by Peters et al. (2015), was applied [29]. In fact, the final version (Peters et al.) constituted the main framework for the design of the present study. Since we sought evidence on the broad topic of the role of political participation in health, the scoping review method was selected to achieve this purpose. The breadth of the main research question, the dispersion of evidence regarding political participation in health, and the flexibility required to align with the conceptual framework used made this method the most appropriate choice. To enhance accuracy and transparency, the various stages of this study were conducted in accordance with the JBI Manual for Evidence Synthesis [30] and the PRISMA‐ScR checklist (Tricco et al., 2018) [31].

According to these guidelines, at least two reviewers are required to independently assess the results of searches and screening; therefore, in this study, two reviewers independently performed this task. In the initial stage of a scoping review, the overall objectives must be specified. While in systematic reviews, deviations from the initial objectives are rare, in scoping reviews, due to their iterative nature and broad conceptual scope, modifications may occur. Accordingly, the present study specifically explores the role of political participation in health by examining citizen science, political medicine, and other forms of public health involvement.

Based on this aim, the study title was determined. According to the general framework of scoping reviews, the title should reflect three main elements: the population under study, the concept, and the context. In the present study, the population was not primary human data but rather published scientific evidence and documents in the field of political participation in health. Since published evidence is a common feature of all scoping reviews, and readers implicitly understand this when encountering the term Scoping Review, the title did not explicitly mention the “population.” Therefore, considering the nature and aim of the study, the title was written with a focus on the concept (political participation) and the context (health).

According to the general criteria for writing scoping reviews, an appropriate overall framework should include the main elements of the subject, key definitions, and the existing body of knowledge in the research field. Based on current knowledge and scientific experience, political participation in health, as one of the key dimensions of health governance, plays a decisive role in shaping policies, enhancing accountability, and increasing transparency [19, 20, 28, 32]. The concept of political participation in health encompasses a wide range of activities, including citizen involvement in decision‐making processes, citizen science, political activism, and participation in local institutions. Given the impact of these issues on the health sector and the necessity of addressing them, they have received increasing attention in recent years. However, the existing evidence is largely scattered and heterogeneous, necessitating a scoping review to map its various dimensions.

In general, in the present study, the key concepts were considered based on definitions related to each dimension. Accordingly, health policy is defined as a set of actions or even inactions that affect organizations, staff, services, financial mechanisms, and all stakeholders in the health sector [2]. Social participation is defined as the process of creating enabling relationships among stakeholders to address issues related to the health sector [33]. Political participation refers to activities through which citizens influence the selection of policymakers or the policies being implemented, while political activism is a form of political participation that often takes place directly, at the public and grassroots level, to shape policy agendas, and is distinct from professional participation in politics [18, 34]. Citizen science is a concept that refers to the active involvement of ordinary people or the general public in scientific processes, whether through collaboration with professional researchers or by independently applying scientific methods [35]. Since this study is a scoping review, it lacks numerical data and statistical results. It has conducted no statistical test, and the analysis followed the JBI methodology and PRISMA‐ScR guidance.

### 3.2 Inclusion and Exclusion Criteria

In line with the general framework of scoping reviews, inclusion and exclusion criteria were defined. The inclusion criteria in this study, similar to systematic reviews, served as a guide for selecting sources, and the rationale for each criterion was explained in the study background. Studies were included if they examined political participation in health. Such participation could occur at different levels, including citizens, local communities, or health professionals. Only works that reported outcomes or factors related to health were included. No restrictions were applied regarding study design (quantitative, qualitative, or mixed‐methods) or study setting, in order to cover a wide range of evidence. However, only studies published in English and Persian were considered. Regarding participants, all age groups and populations were included, provided that their political participation in health was examined.

Exclusion criteria were as follows: studies focusing on animal populations were excluded. Articles without full‐text access or with invalid access links were also excluded. Publications lacking scientific content or relevance to the subject (such as purely visual articles, non‐scientific reports, or sources without analytical data) were also excluded.

### 3.3 Search Strategy and Databases

In accordance with JBI guidelines, a three‐step search strategy was employed. In the first step, a preliminary search was conducted in PubMed and Scopus to identify relevant keywords and indexing terms. At this stage, two reviewers exchanged different search strategies to ensure that relevant studies were not missed while minimizing irrelevant results. In the second step, after finalizing the search strategy, a comprehensive search was conducted in PubMed, ProQuest, Google Scholar, Scopus, and Web of Science on February 19, 2023. Boolean operators (“OR” and “AND”) were used to combine keywords. The main keywords included “citizen science,” “political medicine,” “political participation,” “health politics,” “health care,” “health,” and “political participation determinants.” In the third step, the reference lists of key articles and related reviews were manually checked to ensure comprehensiveness. No time restrictions were applied, but only articles published in English and Persian were included. Search results were stored in EndNote X8.1, and duplicates were removed. Finally, all identified records were screened independently by two reviewers to ensure transparency and accuracy in the selection process.

### 3.4 Screening and Data Extraction

As noted earlier, after importing the data and before starting the screening process, duplicates were removed using EndNote. Based on the inclusion and exclusion criteria, the main topic and objectives guided the screening process, which was conducted in three stages: title screening, abstract screening, and full‐text screening. At each stage, some studies were excluded according to the predefined criteria, and the remaining ones advanced to the next stage. Ultimately, after full‐text screening, a number of studies were included in the final stage (data extraction). For data extraction, the standard form provided by JBI was used. Extracted information from each study included: first author, country and year of publication, study design and subject, target group (citizens, local communities, health professionals, and other stakeholders), influencing factors categorized according to the STEEP analytical framework (social, technological, economic, environmental, political), and the type of reported impact (including health outcomes, civic participation, enhanced accountability, or improved health governance).

### 3.5 Analytical Framework

In this study, the STEEP analytical framework was used to analyze the extracted data, as it is widely applied in policy and futures studies. In recent years, it has also been employed as a dynamic approach for modeling and analyzing sustainable development policies [36]. The STEEP framework generally includes five main dimensions: social, technological, economic, environmental, and political. The social dimension encompasses factors such as public participation, cultural norms, and health literacy. The technological dimension refers to digital health tools for civic engagement. The economic dimension relates to issues such as income inequality, poverty, financial stability, and access to resources. The environmental dimension considers access to services, facilities, and infrastructure. The political dimension includes governance structures, institutional frameworks, opportunities for civic activism, and similar factors.

The findings of the present study were ultimately summarized according to the STEEP framework and categorized into one of these dimensions based on their concepts and content. This approach enhanced the coherence and clarity of the results and provided greater consistency to the data extracted from the reviewed studies. To further structure the analysis, the SWOT^1^ framework was also applied. This framework enabled the identification of strengths, weaknesses, opportunities, and threats related to political participation in health. SWOT analysis was particularly useful in identifying facilitating factors, structural barriers, and potential strategies for strengthening political participation in health processes. During the data analysis stage, the extracted information was thematically organized. The findings were first mapped according to the STEEP^2^ framework to illustrate how each of the social, technological, economic, environmental, and political factors influenced political participation in health. Subsequently, SWOT analysis was used to identify and classify key factors.

### 3.6 Ethical Considerations

Since this study is a scoping review based on published literature, no primary data collection or human subjects were involved. Consequently, ethical approval was not required. By applying a rigorous and structured methodology, this review contributes valuable insights into the determinants and impact of political participation in health, laying the foundation for future research and policy development in this critical area.

## 4 Results

**Figure 1:**
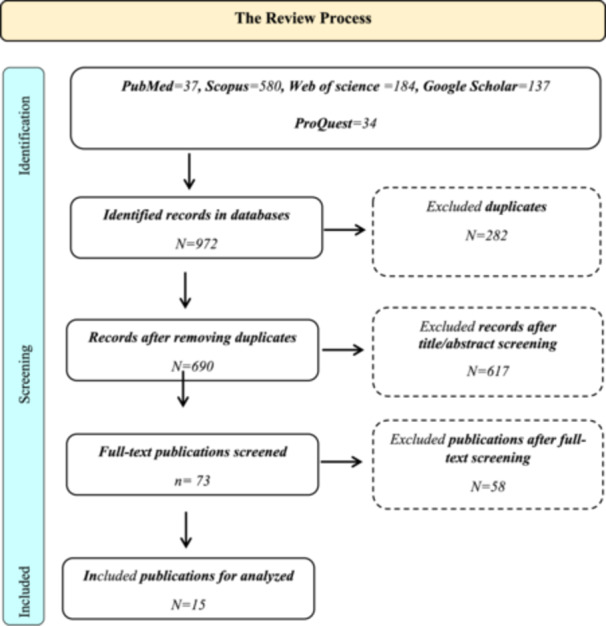
Flow chart of the review process. This Flow chart of the review process “A PRISMA‐ScR flow diagram shows the identification, screening, eligibility assessment, and final inclusion of studies in the scoping review”.

The systematic review process resulted in the identification of 972 records across the databases. After removing 282 duplicates, 690 unique records were screened based on their titles and abstracts. This screening led to the exclusion of 617 records, leaving 73 articles for full‐text review. Following a thorough evaluation of these articles, 58 were excluded for various reasons, such as lack of relevance or insufficient data, resulting in 15 studies being included for detailed analysis (Figure 1).

### 4.1 Summary of Studies

The final 15 studies were diverse in their geographical locations, methodologies, and focal points regarding political participation in health. These studies spanned multiple countries, including the United States, Germany, the United Kingdom, India, Nordic countries, and various regions in Africa, Asia, and Latin America. These studies provide a detailed summary of the included studies, showcasing their study design, year of publication, country, study objectives, target group, influencing factors, and the type of impact observed. These studies utilized various methodologies, including qualitative, quantitative, and mixed‐method approaches, to explore different dimensions of political participation in health (Table 1). Understanding the complex interactions between environmental factors and how they affect political participation in the health sector was also explored using STEEP analysis (Table 2).

**Table 1:** Summary of studies reporting factors of political participatory in health.

Primary Author	Country/Year	Study design/Study objects	Target group	Influencing factors	Type of impact	Participation type
Grant, G.A [32].	**USA**/1989	**Qualitative**/Examine the policy and political implications of consumer participation in health planning	**Health consumers in the United States: 1. Diverse Consumer Groups**: Involves patients, families, and community members from various backgrounds. **2. Engagement Levels**: Varies in levels of engagement from advisory roles to active decision‐making. **3. Health Planning**: Participation in the development and implementation of health policies and programs. **4. Policy Implications**: Impact on health policy development, emphasizing the importance of consumer input. **5. Political Implications**: Examines the political dynamics and power structures influencing consumer participation.	Consumer participation, health planning	Active consumer participation in health decision‐making can lead to improved local responsiveness, increased transparency, and strengthened democracy in health planning processes.	Consumer participation (advisory roles, decision‐making)
Mansfield, D. and Morris [37]	**Peru**/1981	**Mixed Method**/Examine strengths, challenges, and lessons learned in Peru's primary health care system	**Rural and urban populations in Peru:** 1. **Rural Areas**: Limited access to health services, reliance on traditional medicine, lower socioeconomic status. **2. Urban Areas**: Better access to health services, more advanced medical infrastructure, higher socioeconomic status.	Multi sectoral policies, integration of health services, indigenous culture	Strengthening community participation and incorporating culturally appropriate methods can significantly improve health outcomes.	Community participation (local health planning)
Lühr, M., Pavlova, M.K., et al. [28]	**Germany, United Kingdom**/2019	**Qualitative**/Examine the relationship between different types of participation and mental health across age groups	**General population in Germany and the United Kingdom: 1.** Age diversity (young adults, middle‐aged, older adults) **2.** Varied levels of political engagement and volunteerism. **3.** Differences in mental health and social well‐being outcomes	Political versus non‐political participation, mental health, social well‐being	Both political and non‐political participation can influence mental health and social well‐being, with varying effects across different age groups.	Political & non‐political participation (volunteering, civic engagement)
Feldman, C.H, Darmstadt, GL. et al. [20]	**India**/2014	**Qualitative**/Understand how local political economies affect health through women's political participation	**Rural women in India: 1.** Low socioeconomic status **2.** Limited access to health services **3.** Traditional gender roles and cultural norms **4.** Involvement in local governance (e.g., Panchayats)	Political participation, local infrastructure, health systems, cultural norms	Women's political participation influences health by enhancing health agency and access to health resources, leading to better health outcomes in rural areas.	Women's political participation (local governance, Panchayats)
Söderlund, P. and Rapeli, L. [38].	**Nordic countries**/2015	**Qualitative**/Investigate the relationship between personal health and political participation	**General population in Nordic countries: 1.** High levels of political engagement **2.** Comprehensive health care systems **3.** Varied socioeconomic backgrounds **4.** Differences in health status and political activity across age groups	Personal health, socioeconomic status, welfare policies	Poor health can stimulate participation in various political activities, especially those that are time‐consuming and direct, due to high personal stakes.	Political participation (voting, activism)
Serrata, R., Chacur‐Kiss, K., et al. [39]	**Many African, Asian, and Latin American countries**/2020	**Qualitative**/Examine the negative aspects of political participation among older adults	**Older adults: 1.** Age‐related health issues **2.** Varied political engagement levels **3.** Social isolation and mobility limitations. **4.** Experiences of disenchantment or frustration with political processes	Age, political engagement, social factors	Older people's political participation can have negative consequences, such as reinforcing age‐based stereotypes and social exclusion.	Political participation (older adults)
Den Broeder, L., Devilee, J., et al. [40]	**LMICs** * **in Africa, Asia, and Latin America and Specific areas in Europe**/2018	**Qualitative**/Develop a framework for evaluating citizen science projects in public health	**Public health citizen science projects in the Netherlands: 1.** Diverse health topics (e.g., environmental health, chronic disease management) **2.** Varied levels of citizen engagement **3.** Collaboration between citizens and health professionals 4. Impact on public health policies and practices	Monitoring and evaluation methods, citizen participation, public health	A comprehensive evaluation framework can improve the quality and effectiveness of citizen science projects in public health, leading to better community health outcomes.	Citizen science participation
Reeves, A., Johan P. et al. [19]	**Many European countries**/2019	**Qualitative**/Explore the correlation between political participation inequalities and health inequalities	**Citizens of 17 European Countries: 1. Individuals with Different Educational Levels**: The study examines political participation (voting behavior) segmented by educational attainment. **2. Individuals with Various Health Statuses**: Health status is measured through self‐reported health and other health indicators. **3. Different Socio‐Economic Groups**: The analysis includes socio‐economic variables such as income, employment status, and access to health resources.	GDP, income inequality, health spending, social protection spending, poverty rates, smoking	Economic factors influence both political participation and health outcomes.	Voting participation (educational & socioeconomic differences)
Gleason, S. [41].	**India**/2001	**Mixed Method**/Investigate the relationship between female political participation and health in India	**Women in Urban Areas of India:** Socioeconomic Status, Political Participation, Health Outcomes, Cultural and Social Factors **Women in Rural Areas of India**: Socioeconomic Status, Political Participation, Health Outcomes, Cultural and Social Factors	Cultural factors, political participation, social development	Women's political participation can positively impact their health and social well‐being by strengthening their voice and influence in social and political decision‐making.	Women's political participation
Siddiqi, S. M., Uscher‐Pines, L. et al. [26]	**USA**/2019	**Qualitative**/Assess readiness and experiences of health departments with citizen science	**Local health departments in the USA: 1.** Varying levels of resources and capabilities **2.** Diverse geographical locations, including urban and rural settings **3.** Different organizational structures and readiness for citizen science	Knowledge and attitudes, readiness, experiences, barriers	Citizen science can enhance emergency health preparedness and response but requires increased awareness and readiness among local health department staff.	Citizen science participation
Wolkorte, R. and Wildevuur, S. [42].	**Many Africa, Asia, Latin America and Europe countries**/2021	**Mixed Method**/Develop an effective framework for evaluating citizen science projects in health and well‐being	**Citizen science projects globally: 1.** Diverse projects with various health‐related focuses **2.** Different methodologies and tools used for citizen science **3.** Global geographical distribution	Monitoring and evaluation methods, citizen participation, health and well‐being	Creating a comprehensive evaluation framework for citizen science in health can improve project quality and effectiveness, leading to better outcomes for participants and communities.	Citizen science participation
Sun, Y., Graham, T., et al. [43]	**China**/2016	**Qualitative**/Investigate how public health issues are discussed in Chinese BBS forums	**Chinese internet users: 1.** Active participants in online forums **2.** Varied opinions and discussions on public health issues **3.** Influence of internet censorship and government policies **4.** Demographic diversity (age, gender, socioeconomic status)	Public deliberation, civic engagement, online communication	Online discussions can enhance civic engagement and influence public health policy by providing a platform for public deliberation and diverse viewpoints.	Online civic participation (forums, deliberation)
Loh, A., Simon, D. et al. [44]	**Germany**/2007	**Qualitative**/Examine the current state and future perspectives of participation in German healthcare	**Patients and citizens in Germany: 1.** Varied levels of health literacy **2.** Diverse socioeconomic backgrounds **3.** Differences in participation levels between urban and rural areas **4.** Experiences with the German health care system	Healthcare policies, patient involvement	Increasing patient and citizen participation can improve healthcare quality and responsiveness, but challenges such as limited engagement and bureaucratic barriers remain.	Patient participation
L. Beatty, A., Peyser, N.D., et al. [45]	**USA**/2020	**Qualitative**/Investigate the long‐term health impacts of COVID‐19 using a digital cohort	**General population in the United States: 1.** Diverse demographic backgrounds **2.** Varied levels of technology access and literacy **3.** Differences in health behaviors and outcomes related to COVID‐19	Digital health, longitudinal study, COVID‐19	A digital health cohort study can provide valuable insights into the long‐term effects of COVID‐19 and inform public health responses and policies.	Digital participation (online health cohort)
M. McGuire, C., Gollust, S.E, et al. [46]	**USA**/2021	**Qualitative**/Investigate health as a resource for political participation among low‐income workers	**Low‐income workers in two cities in the United States: 1.** Low‐income status **2.** Employment in low‐wage jobs 3. Barriers to political participation (e.g., health issues, time constraints) **3.** Experiences with local political processes and voting behavior	Health status, political participation, socioeconomic factors	Health plays a key role in political participation, as healthier individuals are more likely to vote and engage in politics, emphasizing the need for policies that reduce health disparities to boost civic involvement.	Political participation (voting, local processes)

* Low‐ and middle‐income countries.

**Table 2:** STEEP framework for factors of political participatory in health.

Category	Factor	Determinant
Social	Public Participation	Local engagement, Community involvement, Citizen participation, Consumer participation, Community roles, Patient involvement, Civic engagement, Stakeholder engagement
	Cultural Factors	Women's rights, Indigenous culture, Public trust, Community‐driven initiatives, Public awareness, Staff readiness, Educational background, Citizen science
	Social Status	Social inclusion, Social development, Job stability
	Health Factors	Personal health, Health status, Public health engagement, Health awareness, Social well‐being, Health inequalities, Community health resources, Integration of health services, Health agency, Public health issues, Smoking
	Educational	Knowledge and attitudes, Educational attainment, Health literacy
Economic	Spending Status	Social protection spending, Health spending
	Income Status	Income levels, Income inequality, Poverty rates, GDP
Technological	Digital Tools	Data collection, Data analysis, Feedback mechanisms, Monitoring and evaluation methods, Digital collaboration, Longitudinal data collection, Mobile health technologies, Online discussions
Environmental Political	Place	Local infrastructure
	Planning	Women's voice in decision‐making, Political participation, Policy‐making, Democracy, Health planning, Public health policies, Multi‐sectoral policies, Local governance, Government response, Policy implementation, Healthcare policies, Civic engagement, Public policies

### 4.2 Social and Cultural Determinants

Social determinants, such as public participation and social inclusion, play a critical role in enhancing political participation in health. Strategies to promote civic engagement and reduce social exclusion are essential for effective health governance. Cultural factors, including indigenous practices and community‐driven initiatives, must be recognized and integrated into health policies to improve their acceptance and effectiveness. Cultural norms about trust in governmental institutions profoundly affect citizens' readiness to engage in health‐related decision‐making. Moreover, disparities in community health literacy and shared norms of preventive activities influence participation trends across diverse demographic groups.

Socio‐economic determinants—including income inequality, resource distribution, and access to participatory infrastructures—emerged as major factors influencing civic engagement in health. Health literacy also played a crucial role within this socio‐economic context. As highlighted in the results, limited health literacy co‐occurred with lower levels of political participation, reduced trust in institutional communication, and diminished capacity to navigate digital health platforms. This finding is consistent with prior research demonstrating that health literacy is a core socio‐economic determinant that shapes individuals' ability to engage effectively with health systems (Table 2).

### 4.3 Technological and Environmental Determinants

Leveraging digital tools for data collection and feedback enhances the monitoring and evaluation of health policies, facilitating broader participation. In this study, environmental factors are defined as the broader societal, institutional, and policy contexts that influence public participation in health governance, as opposed to the natural or ecological environment.

Investing in local health infrastructure supports better access to health services and encourages community involvement (Table 2).

### 4.4 Economic Determinants

Policies aimed at reducing income inequality and poverty are vital for enhancing political participation in health. Economic stability enables individuals to engage more effectively in political processes and advocate for their health needs (Table 2).

The SWOT analysis was done to bring together the most important internal and external aspects that affect public engagement in health policy. This made it possible to systematically look at the existing governance landscape's strengths, weaknesses, opportunities, and threats.

**Figure 2:**
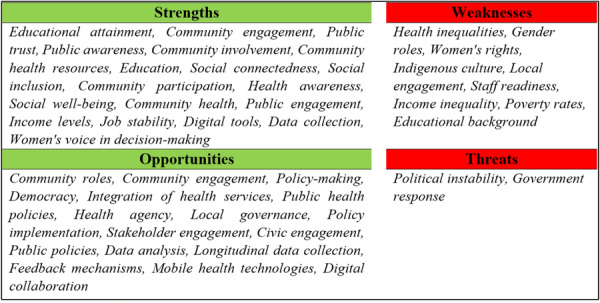
SWOT Analysis, it is a schematic summary of political participation in health “including strengths, weaknesses, opportunities, and threats which influence political participation in health, based on extracted evidence.” *Note:* The four fields show how civic engagement mechanisms interact with social, technological, economic, and policy‐related issues. They show the interaction of political involvement dimensions and public health determinants.

Based on content analyses of the extracted qualitative materials on SWOT analysis shows that the intervention of political participation in health can significantly improve health outcomes and democratic governance. Therefore, by eliminating threats, correcting weaknesses and using opportunities, the role of political participation in health can be increased (Figure 2).

### 4.5 Strengths and Opportunities

Active community engagement in health planning has emerged as a key strength, enhancing local responsiveness and transparency. Educational attainment plays a crucial role; as higher levels of health literacy enable individuals to participate more effectively in health‐related political activities. Additionally, the use of digital tools and mobile health technologies facilitates broader participation and improves the collection and analysis of health data.

Opportunities lie in integrating political participation into health policies, which can strengthen community roles and ensure health services align with the population's needs. Engaging a diverse range of stakeholders, particularly marginalized groups, can lead to more inclusive and comprehensive health policies. Promoting civic engagement through education and awareness campaigns empowers communities to actively participate in health governance, fostering sustainable health improvements.

### 4.6 Weaknesses and Threats

Persistent health inequalities, driven by socioeconomic disparities, remain a significant weakness. Communities with lower socioeconomic status often lack the resources and access necessary for effective political participation. Cultural barriers, such as traditional gender roles and norms, restrict women's political participation, limiting their influence on health policies and outcomes. Variations in educational attainment also affect individuals' ability to engage meaningfully in health politics.

Threats include political instability, which can deter participation and disrupt health initiatives. Ineffective or delayed government responses to health crises can erode public trust and discourage community involvement in health decision‐making. High levels of income inequality and poverty further hinder political participation, as individuals prioritize immediate survival concerns over political engagement.

## 5 Discussion

Inclusive political participation plays a vital role in achieving equitable and sustainable health improvements. Understanding the factors shaping political engagement in health provides valuable insights for improving health outcomes and strengthening democratic governance within health systems. This review highlights the complex interplay of social, economic, political, and cultural influences that shape health‐related political participation. These technological advancements improve access to information and facilitate virtual participation, empowering individuals to take part in health‐related decision‐making.

It is also important to recognize that promoting participation occurs within systems that face structural and political constraints. Policymakers often operate under limited financial resources, staff shortages, and competing policy agendas, which may reduce incentives to engage citizens in decision‐making processes [36]. Additionally, institutional path dependencies and political considerations can discourage the adoption of participatory mechanisms, particularly when they challenge existing power dynamics or extend decision‐making timelines [47, 48]. These systemic barriers illustrate that fostering participation requires not only community‐level capacity building but also structural reforms that enable meaningful engagement decisions to take longer [47, 48]. These systemic impediments show that encouraging participation needs both building capacity at the community level and changes to the system that make it possible for people to get involved in a meaningful way.

Even with these problems, policymakers may still find it helpful to use participatory methods. Evidence indicates that inclusive governance can bolster policy legitimacy, boost accountability, diminish implementation resistance, and produce solutions that are more culturally relevant [7, 49]. Participatory methods can also help people and institutions trust one other more, which is very crucial during health emergencies and policy changes. So, even while institutional barriers make it harder for people to take part, the possible benefits for successful governance are a solid reason for politicians to put money into processes that include everyone.

The intersection of socioeconomic status and health literacy remains critical. Targeted education programs aimed at enhancing health literacy help mitigate the effects of socioeconomic disparities, allowing individuals to participate more effectively in health policymaking [50]. Higher health literacy enables people to better understand policies, engage in discussions, and advocate for their communities' health needs. Political participation also influences mental health. Recent investigations suggest that involvement in political decision‐making fosters a sense of purpose and belonging, leading to improved mental well‐being [51]. When individuals feel their voices matter in shaping health policies, their overall psychological and emotional health benefits.

Despite these advantages, barriers to effective participation persist. Socioeconomic inequalities, cultural norms, and inadequate infrastructure hinder civic engagement in health policy. Feldman et al. pointed out that traditional gender roles and limited healthcare access significantly restrict women's political participation in rural India [20]. Addressing these barriers requires comprehensive interventions, including community‐driven initiatives that integrate health services with civic education [52]. The review also revealed key insights into the factors influencing political participation in health. Socioeconomic status and health literacy significantly shape engagement, with McGuire et al. finding that healthier individuals are more likely to participate in political processes, reinforcing the need to reduce health disparities [46]. Barriers such as social exclusion and limited access to political processes were evident, particularly in Feldman et al.'s study on rural India [20]. Studies also demonstrated the positive effects of political participation on health outcomes, with Lühr et al. showing that engagement in political and non‐political activities influences mental health and social well‐being [28].

**Figure 3:**
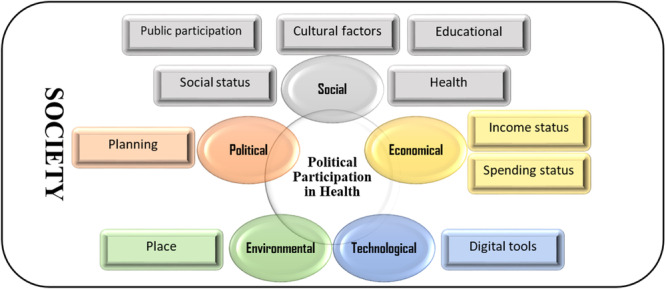
Conceptual modeling relationships between categories, STEEP framework, this conceptual model illustrates how social, technological, economic, environmental, and political factors interact with political participation in health.

Active community involvement in health governance leads to more responsive and transparent health policies. Grant et al. and Mansfield & Morris illustrated that diverse consumer groups participating in health planning enhance local accountability and democracy in health processes [32, 37]. Additionally, Den Broeder et al. emphasized that well‐structured citizen science projects contribute to public health policy improvements and community well‐being [40]. Figure 3 presents the conceptual relationships between political participation, health determinants, and environmental influences through the STEEP framework. By promoting health literacy, ensuring equitable access to political processes, and addressing socioeconomic disparities, policymakers can develop inclusive and effective health strategies. The results above are similar to these findings. In the STEEP dimensions, socio‐political factors were the most common (27 comments), followed by economic barriers (22 statements) and technology facilitators (15 statements). The significance of these categories in the content analysis highlights their pivotal role in influencing public engagement in health governance.

It appears that the perspectives of international organizations and institutions such as WHO, ILO, and the United Nations are consistent with the findings of the present study, which have been derived from existing research on political participation in the health sector [16, 17, 35]. Grant [32] and Mansfield & Morris [37] reported the positive effects of consumer and community participation on transparency, accountability, and cultural appropriateness in health planning. Similarly, the World Health Organization, in its 2024 World Health Assembly resolution, emphasized the need to strengthen social participation in decision‐making across all areas. Moreover, insights from the reviewed studies indicate that both political and non‐political participation influence various dimensions of health across different age groups. Lühr et al. [28] demonstrated that participation in the health sector provides significant benefits, particularly in terms of psychological well‐being and social welfare. Therefore, participation is not only a necessity but also a determinant of health that requires greater attention.

The findings of the present study suggest that increasing political participation in health may lead to more transparent and accountable health governance. Furthermore, medicine and nursing within the health sector may be directed toward greater attention to patient‐centered needs and individual preferences. In addition, enhanced political participation in health appears to clearly support the advancement of collective, democratic, and social policies aimed at reducing inequalities and promoting social equity. Therefore, adopting policies that facilitate participation, reduce barriers, and strengthen local capacities is essential for building inclusive and responsive health systems. Achieving these goals seems to require improving health literacy, reducing barriers to participation, and creating opportunities for active involvement in health, a process that can be facilitated through digital tools. Recent literature reinforces the importance of political participation in shaping health policies. Reducing barriers and leveraging technological advancements can increase civic engagement in health governance. Continued research and targeted interventions are crucial to understanding the mechanisms through which political participation impacts health, guiding the development of more effective policies. These results show how important it is to make policies that lower structural barriers, improve local capacities, and make forms of involvement a part of health governance systems.

## 6 Conclusion

This study examines the role of political participation in health by analyzing scientific literature on citizen science, political medicine, and other forms of public health engagement. Political participation in health appears to be more than a civil right, as it functions as a significant determinant of health outcomes at both individual and community levels. Active involvement of citizens, particularly among groups such as rural women, older adults, and economically disadvantaged populations, can contribute to advancing health equity, which is recognized as both a human right and a core objective of health systems. The findings of this study indicate that social, economic, technological, environmental, and political factors influence the scope and quality of participation. Domains such as income, education, and health status, which are reflected through mechanisms like literacy, access to services, and public awareness, can shape the nature of political engagement in health. Among these, social determinants of health appear to be among the most influential drivers of participation, especially in contexts where community involvement and civic engagement are central.

Political participation in health is not merely a tool for achieving sectoral goals but represents a foundational mechanism for realizing democratic governance in health. Therefore, adopting policies that facilitate participation, reduce barriers, and strengthen local capacities is essential for building inclusive and responsive health systems. It is also recommended that future research quantitatively examine the impacts of political participation on health outcomes so that more precise and actionable insights can be generated.

### 6.1 Limitations

In the present study, only publications in English or Persian were included; therefore, if valuable evidence exists in other languages, it was not captured in this review. Since scoping reviews primarily aim to explore different dimensions of a topic, and the findings of this study were mostly qualitative or mixed‐method in nature, the absence of quantitative and comparative data makes generalization of the results more difficult.

Furthermore, many of the included studies did not clearly report their sampling procedures, with several relying on convenience or purposive samples, which introduce potential selection bias and limit the representativeness of their findings. The methodological diversity—ranging from descriptive qualitative designs to mixed‐method case studies—also restricts the comparability of results across settings. In addition, because the review excluded gray literature and studies published in languages other than English and Persian, relevant evidence from low‐resource or non‐Anglophone regions may be underrepresented. These factors collectively constrain the generalizability of the findings and highlight the need for more rigorous and diverse empirical research.

Moreover, given the limited number of studies conducted in this area, the collected evidence comes from countries with diverse socio‐economic and political contexts, which complicates direct comparison and conclusion. As no primary data were collected and the focus was on existing studies and reports, the quality of the results is inherently dependent on the quality and accuracy of those sources.

## Author Contributions

**Morteza Arab‐Zozani:** conceptualization, writing – original draft, methodology, investigation, writing – review and editing, data curation, resources. **Marzie Zarqi:** conceptualization, writing – original draft, methodology, writing – review and editing, data curation.

## Funding

The authors have nothing to report.

## Conflicts of Interest

The authors declare no conflicts of interest.

## Transparency Statement

The lead author Zahra Heydarifard, Majid Heydari affirms that this manuscript is an honest, accurate, and transparent account of the study being reported; that no important aspects of the study have been omitted; and that any discrepancies from the study as planned (and, if relevant, registered) have been explained.

## Data Availability

The authors have nothing to report.

## Endnotes

1 SWOT: (Strengths, Weaknesses, Opportunities, and Threats)

2 STEEP (Social, Technological, Economic, Environmental, and Political) framework
